# Transanal opening of intersphincteric space (TROPIS) treatment for high complex anal fistula: a systematic review and meta-analysis

**DOI:** 10.1097/JS9.0000000000002880

**Published:** 2025-07-09

**Authors:** Pengfei Zhou, Jingen Lu, Yanting Sun, Jiawen Wang

**Affiliations:** a Department of Anorectal Surgery, The First Affiliated Hospital of Henan University of CM, Zhengzhou, Henan, China; b Department of Anorectal Surgery, Longhua Hospital, Shanghai University of Traditional Chinese Medicine, Shanghai ,China

**Keywords:** anal fistula, high complex anal fistula, meta-analysis, systematic review, transanal opening of intersphincteric space (TROPIS)

## Abstract

**Background:**

The transanal opening of intersphincteric space (TROPIS) is a new surgical modality for treating anal fistulas. This procedure involves a transanal incision between the sphincters to promote drainage, excision of the infected areas, and maintenance of sphincter patency. In this meta-analysis, we evaluated the efficacy and safety of TROPIS in treating high complex anal fistula.

**Methods:**

We searched the PubMed (NLM), LISTA (EBSCO), and Web of Science Core Collection (Clarivate) databases for all related clinical research literature on highly complex anal fistulas treated with TROPIS from the time of database creation to 31 December 2024, with inclusion restricted to studies published in English. All included articles follow the PICO principle. Data on surgical one-time cure rate, recurrence rate after the first surgery, final cure rate, and occurrence of adverse events were extracted for further analysis.

**Results:**

Six studies involving 499 patients were included. The heterogeneity test revealed no heterogeneity, and results for one-time cure rate showed I^2^ = 0.00% < 50%, and Q test *P* = 0.81 > 0.1 (effect size [ES] = 0.80, 95% confidence interval [CI] (0.77, 0.84), Z = 45.96, *P* = 0.00), where the one-time cure rate was 80%; recurrence rate after first surgery I^2^ = 0.00% < 50%, and Q test *P* = 0.81 > 0.1 (ES = 0.20, 95% CI (0.16, 0.23), Z = 17.49, *P* = 0.00), and the recurrence rate is 20%; final cure rate I^2^ = 0.00% < 50%, and Q test *P* = 0.82 > 0.1 (ES = 0.89, 95% CI (0.86, 0.92), Z = 51.21, *P* = 0.00), and the final cure rate was 89%. No adverse events were reported in any of the patients. Sensitivity analyses showed excellent stability of the surgical cure results in all studies. No publication bias was detected in any of the included studies.

**Conclusions:**

The TROPIS procedure for highly complex anal fistulas exhibits a high one-time cure rate, low recurrence rate, and high overall cure rate and is not associated with any adverse events.

## Introduction

Anal fistula is a common colorectal-anal disease, and anal gland infection is the primary factor in anal fistula development[1]. Anal fistula formation is typically caused by infection of the anal glands between the sphincters[2]. Removal of the primary infected foci is a crucial step in anal fistula treatment because fistula formation reduces the likelihood of spontaneous healing and necessitates surgical intervention for complete disease eradication. During anal fistula surgery, focusing on protecting the sphincter muscle and anal function, as well as the cure rate of the chosen surgical procedure, is crucial in improving the patient’s quality of life[3]. Furthermore, the location and nature of the fistula tube in highly complex anal fistulas make it extremely easy for patients to be misdiagnosed and mistreated, leading to the recurrence of perianal symptoms. Highly complex anal fistulas have a high recurrence rate and greater risk of complications (such as fecal incontinence), thereby posing notable challenges to both affected patients and their doctors[4]. In addition, gold standards and satisfactory treatment methods are lacking for highly complex anal fistulas[5]. Currently, the traditional method of cutting setons has demonstrated a therapeutic effect on these fistulas. However, this technique involves cutting or strangling the anal sphincter, leading to scarring and an escalation of the risk of anal incontinence and other postoperative complications[6]. Along with the conventional resection of anal fistulas, procedures such as ligation of the intersphincteric fistula tract (LIFT)[7] and transanal opening of the intersphincteric space (TROPIS)[5] are increasingly being incorporated into clinical practice to preserve the sphincter muscle. Furthermore, new technologies such as the anal fistula plug, video-assisted anal fistula treatment (VAAFT), endorectal advancement flap, and over-the-scope clip are being widely employed in clinical settings[8]. These surgical techniques have shown promise in safeguarding anal function, enhancing surgical success rates, and lowering the risk of anal orifice recurrence and the need for multiple surgeries. However, the inherent novelty of these treatment modalities and the limited body of supporting research have led to a scarcity of strong evidence supporting these new techniques[9].

Colorectal and anal surgeons focus on improving the success rate of anal fistula surgery through advancements in medical technology with the goal of reducing recurrence, mitigating postoperative complications, and ultimately preserving the normal physiological function of the anal sphincter. TROPIS is a new surgical procedure introduced by Garg, who first applied this approach for the treatment of complex anal fistulas in 2017[10]. Compared with other surgical treatments, TROPIS involves a transanal incision of the intersphincteric space to promote drainage, excise infected tissue, and sustain sphincter patency to enhance healing by secondary intention.

In this study, we aimed to assess the efficacy of TROPIS in managing highly complex anal fistulas, with a specific focus on the surgical one-time cure rate, recurrence rate after the first surgery, final cure rate, and adverse events. We believe that these findings will offer empirical support for the effectiveness of the TROPIS procedure in treating highly complex anal fistulas. This article is compliant with the TITAN Guidelines 2025—governing declaration and use of AI[11].

## Method

This work has been reported in line with [12]Preferred Reporting Items for Systematic Reviews and Meta-Analyses and[13] Assessing the Methodological Quality of Systematic Reviews Guidelines. The protocol for the study was registered in PROSPERO (Identifier: CRD42023481797).

### Search strategy

In this meta-analysis, the PubMed (NLM), LISTA (EBSCO), and Web of Science Core Collection (Clarivate) databases were searched from the time of database creation to 31 December 2024, to retrieve all related clinical research literature on the application of TROPIS for treating anal fistulas. Only the studies published in English were included. Search keywords include the following: “Anal Fistula” AND “TROPIS”; “Anal Fistula” AND “Transanal Opening of Intersphincteric Space”; “High Complex Anal Fistula” AND “Transanal Opening of Intersphincteric Space”; “High Complex Anal Fistula” AND “TROPIS.” The search keywords comprised a combination of subject and free words, including the full and abbreviated names of the procedure.

### Literature screening and data collection

Two authors (Pengfei Zhou and Jiawen Wang) initially screened the titles and abstracts of the retrieved articles, followed by further searching for eligible literature. The collected data included the name of the first author, publication year, mean patient age, diagnostic modality, number of patients lost or excluded, final number of patients included, surgical modality, study outcome, and follow-up duration.

**HIGHLIGHTS**

- Transanal opening of intersphincteric space is a new surgical modality for anal fistula.
- TROPIS promotes drainage and excises infected areas.
- Effectiveness and safety of TROPIS in the treatment of high complex anal fistula.
- No adverse events were reported in any of the patients
- TROPIS exhibits a high cure rate and low recurrence rate.

### TROPIS implementation method

**Figure 1.**
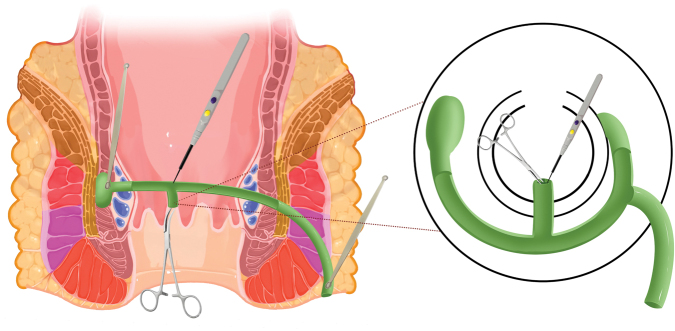
TROPIS implementation method

In TROPIS surgery, the mucosa and internal sphincter on the intersphincteric tract are incised using electrocautery, which destroys the infected crypt gland and opens up the infection pathway in the intersphincteric space, thereby achieving secondary intention. Secondary intention is a natural healing process of the surgical wound in an open state, not a direct closure by suturing. TROPIS removes the sepsis of both external sphincters without causing any damage to the external sphincters, resulting in good healing on both sides. For horseshoe shaped fistula, the incision extends to both sides of the midline posterior inner opening. The external opening is slightly enlarged, generally less than 1 cm, and all fistula lesions are thoroughly scraped off (Fig. 1).

### Selection criteria

The inclusion criteria were as follows: 1. Clinical studies published in English. 2. All patients were unambiguously diagnosed with highly complex anal fistulas, including high cryptoglandular fistula-in-ano, horseshoe, and supralevator fistulas. 3. The TROPIS procedure was performed in all patients. 4. The outcome indices included the number of patients successfully treated with surgical interventions.

The exclusion criteria were as follows: 1. Studies on nonsurgical treatments. 2. Studies that employed combined surgical approaches. 3. Studies that did not include highly complex anal fistulas. 4. Studies that did not provide a clear preoperative diagnosis of highly complex anal fistulas. 5. Studies that were not completely available. 6. Duplicate publications containing the same information. 7. These included case reports, reviews, systematic evaluations, and expert opinions. 8. Studies based on animal experiments. 9. Studies enrolling patients with Crohn’s. 10. No officially published studies.

### Quality assessment

Because the included studies were all non-randomized controlled studies, we assessed the quality of all studies using the first eight components of the MINORS quality assessment tool[14]. The Newcastle–Ottawa scale (NOS) was used to evaluate the four prospective studies included in this review. The NOS fully considers the commonalities and differences between different designs and assesses literature quality in terms of three aspects: selection, comparability, and outcomes. The use of this evaluation scale can lead to the development of a clearer and more complete study[15]. The JBI Critical Appraisal Checklist was used to evaluate the quality of the remaining two retrospective studies. This tool facilitates the grading of different design types and individual studies to reflect the plurality of the evidence[16].

### Data analysis

Data analysis was conducted using Stata 18.0 software[17], with interval estimation using 95% confidence intervals (CI) as indicators of impact. Heterogeneity in the results was assessed using Cochran’s Q test combined with I^2^. A fixed-effects model was used in the absence of heterogeneity (*P* >0.1, I^2^ <50%), and a random-effects model was used in the presence of heterogeneity (*P* < 0.1, I^2^ > 50%). In addition, a sensitivity analysis was performed to evaluate the stability and reliability of the results. Publication bias was examined by drawing funnel plots and performing Begg’s and Egger’s tests.

## Results

### Study selection

**Figure 2.**
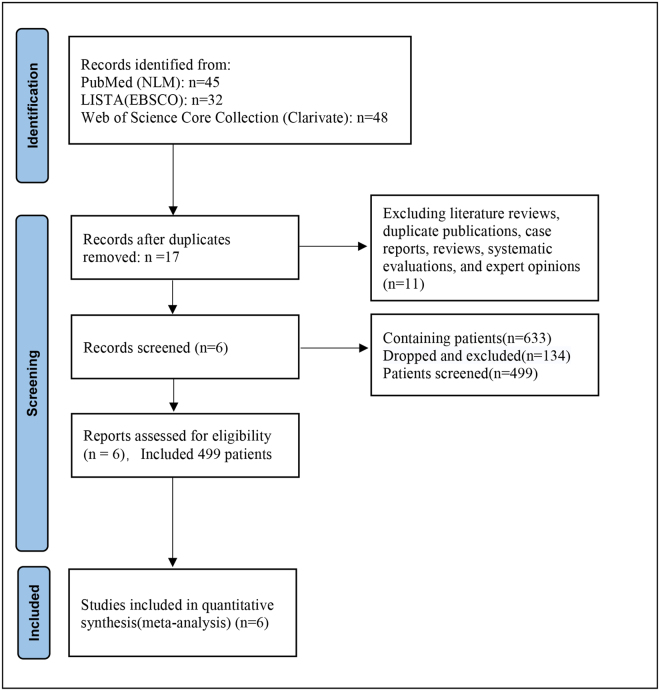
Literature screening flow chart.

After searching the databases, 125 articles were retrieved, including 45 from PubMed (NLM), 32 from LISTA (EBSCO), and 48 from the Web of Science Core Collection (Clarivate). Duplicate screening and comparisons yielded 17 academic publications. After reviewing and excluding studies, six (including a total of 633 patients) were deemed eligible. Of the six included studies, the study period ranged from 2017 to 2024 and included 531 patients, with 499 cases eventually completing the study and 32 failing to complete it. The diagnostic modalities used in all patients included magnetic resonance imaging (MRI) and endoanal ultrasound (EAUS), and all surgical procedures were TROPIS for highly complex anal fistulas. After assessing the literature and excluding patients lost and excluded from the study, 499 patients were finally eligible for inclusion. The selection process for the studies is illustrated in Figure 2, and the details of each included study are presented in Table 1.

**Table 1:** Basic characteristics included in the study

Study	Year	Total case	Gender (male/female)	Average age (years)	Diagnostic methods	Falling off and elimination	Final compliance	Operation	Follow-up time(months)
Yu-bo Li[18]	2022	41	35/6	38.60 ± 13.20	MRI	4	37	TROPIS	6-23
Pankaj Garg[19]	2020	325	292/33	39.90 ± 10.90	MRI	19	306	TROPIS	7–67 (median 36)
Pankaj Garg[20]	2021	21	20/1	Not provided	MRI	0	21	TROPIS	2–62 (median 29)
Pankaj Garg[10]	2017	61	59/2	42.30 ± 9.50	MRI	9	52	TROPIS	6-21
Shrivats Mishra[8]	2024	35	30/5	33.32 ± 10.52	MRI	0	35	TROPIS	3
Baolei Huang[21]	2021	48	41/7	40.00 ± 11.70	EAUS or MRI	0	48	TROPIS	12 (median)

### Quality assessment of included studies

Evaluate the quality of all literature using the MINORS quality assessment tool. At the same time, the NOS was used to evaluate the literature quality of the four prospective studies, and the JBI Critical Appraisal Checklist was used to evaluate the literature quality of the two retrospective studies. The results are summarized in Table 2.

**Table 2:** Quality assessment of included studies

Study	A	B	C	D	E	F	G	H	I	J	Total	MINORS	
Newcastle–Ottawa Scale (NOS)													
YU-bo Li	2	2	2	2	2	0	0	2	2	2	16		14
Pankaj Garg 2020	2	2	2	2	2	0	0	2	2	2	16		14
Pankaj Garg 2017	2	2	2	2	2	0	0	2	2	2	16		14
Shrivats Mishra	2	2	2	2	2	0	0	2	2	2	16		14
JBI Critical Appraisal Checklist													
Pankaj Garg 2021	1	0	1	1	0	1	1	1			6		14
Baolei Huang	1	0	1	1	0	1	1	1			6		14

### Heterogeneity test

**Figure 3.**
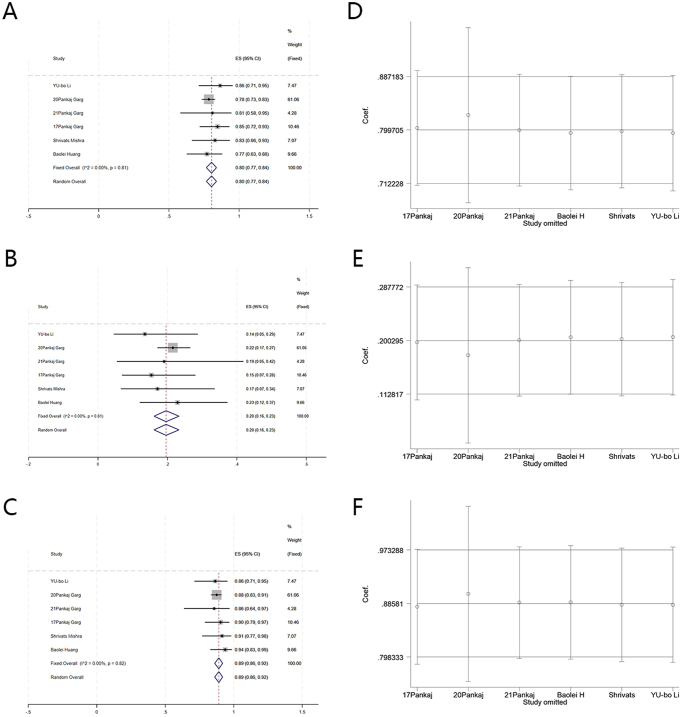
Forest plot of one-time cure rate by surgery, recurrence rate after first surgery, and final cure rate and the results of sensitivity analysis. A. Forest map of one-time cure rate by surgery. B. Forest map of recurrence rate after first surgery. C. Forest map of final cure rate. D. Sensitivity analysis of surgery for a one-time cure rate. E. Sensitivity analysis of recurrence rate after first surgery. F. Sensitivity analysis of final cure rate.

Statistical analyses were conducted for all six included studies. Heterogeneity tests were performed to determine the surgical one-time cure, recurrence after the first surgery, and final cure rates. Surgical one-time cure rate with I=0.00% < 50%, and Q test P = 0.81 > 0.1 (effect size [ES] = 0.80, 95% CI [0.77, 0.84], Z = 45.96, *P* = 0.00); recurrence rate after first surgery with I=0.00% < 50%, and Q test P = 0.81 > 0.1 (ES = 0.20, 95% CI [0.16, 0.23], Z = 17.49, *P* = 0.00), final cure rate with I=0.00% < 50%, and Q test P = 0.82 > 0.1 (ES = 0.89, 95% CI [0.86, 0.92], Z = 51.21, *P* = 0.00). These results indicated that no heterogeneity in all the included literature data on the three outcome parameters, implying good data homogeneity in the literature included in the present study. Consequently, a fixed-effects model was used to analyze both datasets (Fig. 3), followed by a sensitivity analysis to ensure the accuracy and stability of the study findings.

### Sensitivity analysis

The significance of sensitivity analysis lies in evaluating the robustness and reliability of research results, by examining the impact of key hypotheses or method choices on the overall conclusion. Sensitivity analysis for the surgical one-time cure rate, recurrence rate after the first surgery, and final cure rate across the six included studies demonstrated that none of the studies notably interfered with the results of the current meta-analysis, underscoring the excellent stability of this study (Fig. 3).

### Publication bias

**Figure 4.**
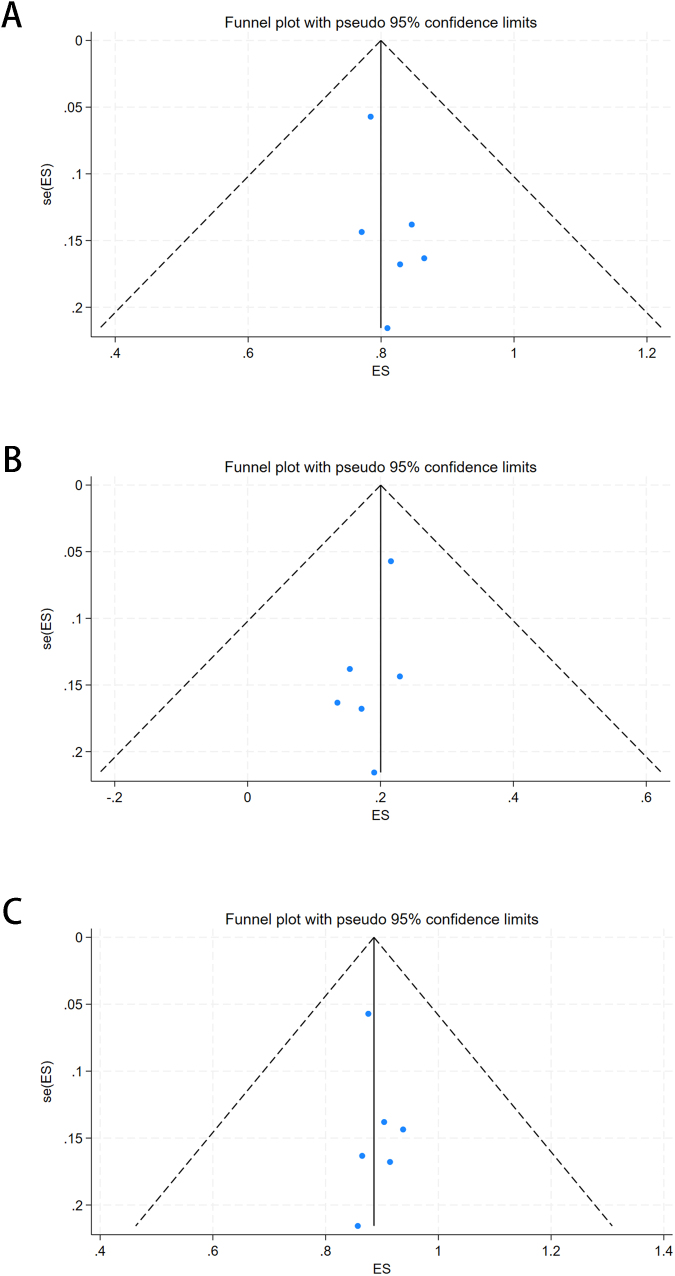
Funnel plot of publication bias. A. Funnel plot of surgery for a one-time cure rate. B. Funnel plot of recurrence rate after first surgery. C. Funnel plot of final cure rate.

**Figure 5.**
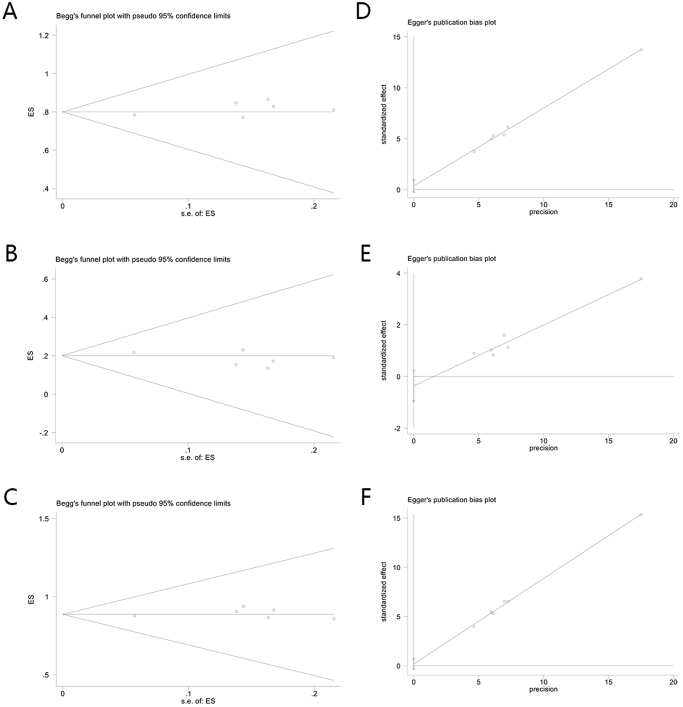
Begg’s test plot of publication bias. A. Begg’s test plot of surgery for a one-time cure rate and Publication bias Egger’s test plots. B. Begg’s test plot of recurrence rate after first surgery. C. Begg’s test plot of final cure rate. D. Egger’s test plot of surgery for a one-time cure rate. E. Egger’s test plot of recurrence rate after first surgery. F. Egger’s test plot of final cure rate.

Funnel plots, Begg’s test, and Egger’s test were used to determine the presence of publication bias. Figure 4 presents funnel plots of the cured patients. The symmetry of the funnel plots indicates no publication bias. A symmetry test of the funnel plot yielded *P* = 0.573 > 0.05, surgical one-time cure rate *P* = 0.707 > 0.05, recurrence rate after the first surgery *P* = 0.851 > 0.05 for final cure rate, highlighting the symmetry of the funnel plot. Additionally, Begg’s test yielded *P* = 0.573 > 0.05 for surgery for a one-time cure rate, *P* = 0.707 > 0.05 for recurrence rate after first surgery, and *P* = 0.851 > 0.05 for final cure rate, as displayed in Figure 5; Egger’s test yielded *P* = 0.573 > 0.05 for surgery for a one-time cure rate, *P* = 0.707 > 0.05 for recurrence rate after first surgery, and *P* = 0.851 > 0.05 for final cure rate (Fig. 5). Therefore, these results indicate that no publication bias existed in the literature included in the present study. Moreover, these findings suggest that TROPIS has significant efficacy in the treatment of high complex anal fistulas in terms of a higher surgical one-time cure rate, final cure rate, and cure rate at the first surgery as well as a lower recurrence rate.

## Discussion

Anal fistulas, which account for 1.7%–2.6% of anorectal diseases, can occur in any age group, with a predominance in middle-aged men, followed by infants and young children[22]. Anal fistulas can be caused by numerous factors, including perianal abscesses, rectal and anal injuries, repeated infections of the anal fissures, various intestinal ulcers, and inflammation[23]. Because of the specific location of anal fistulas and the lack of patient initiative in seeking medical treatment during the early stages of the disease, the symptoms of anal fistulas can recur and even progress to a highly complex anal fistula. Surgical intervention remains the primary treatment for anal fistulas[24]. Low anal fistulas are relatively simple to treat and fistula excision can achieve a high cure rate and postoperative recovery of anal function[25]. In contrast, the direct incision approach for highly complex anal fistulas is accompanied by a higher recurrence rate and damage to anal function. Therefore, accurate preoperative decision making is critical for the successful management of highly complex anal fistulas.

TROPIS emphasizes open drainage of the sphincter space and incorporates two core points: opening of the sphincter space and fistula management. In this procedure, part of the internal sphincter is severed via the anus, allowing the opening of the sphincter gap, removal of the primary infected foci between the sphincters, and drainage maintenance at the trauma site until healing occurs. Moreover, accurate identification of the internal opening in the clinical setting is imperative for successful TROPIS, although the potential risk of overlooking some of the finer curved tubes when addressing the external opening of the fistula also exists in this procedure.

During TROPIS, the deep posterior intersphincteric space (DPIS) opens without disconnecting from the external sphincter. Garg suggested that the foci of infection in the sphincter space are similar to abscesses in a confined space and emphasized that only opening them up and adequately draining them can effectively eradicate the infection and improve the cure rate of anal fistulas. The transanal incision of the intersphincteric space in the TROPIS not only destroys the infected crypt glands but also opens the intersphincteric fistula tract. Consequently, the closed intersphincteric space is converted into an open wound and adequate drainage is achieved to keep the wound open. In contrast to other surgical procedures that require primary treatment to attain a final cure outcome, TROPIS aims to achieve a final cure with a secondary intention. During TROPIS treatment of infected fistulas, draining all pus and ensuring continuous drainage yields a higher rate of complete fistula healing after wound healing[26]. Garg also stressed the need to adequately scratch the fistula wall during TROPIS to achieve proper drainage and preserve open drainage until complete healing. Therefore, maintaining open drainage in the trauma area after fistula surgery is critical for a successful surgery. Although TROPIS widens the internal opening and potentially aggravates the disease by facilitating fecal matter entry into the incision, the impact of these conditions is mitigated because the external anal sphincter does not widen. In most cases, the tension of the external sphincter prevents the fecal material from entering the incision[5]. Furthermore, minimal damage to the external sphincter occurs during the TROPIS procedure, thereby eliminating the risk of fecal incontinence. Finally, considering that TROPIS does not require cutting of the external sphincter, the function of the anus is protected even in cases of infection between the sphincters spreading to the external sphincter.

Landmark studies by Parks and Eisenhammer refined and summarized the glandular infection theory of anal fistulas and established Parks’ classification system, which has become the standard for the clinical staging of anal fistulas and is widely applied in clinical practice^[27,28]^. The treatment of adenogenic anal fistulas comprises of three essential components: management of the primary focus of infection (i.e., the sphincter space), complete excision of the fistula, and drainage patency of the surgical wound. Anal fistulas arising from infection of the sphincter space are similar to closed abscesses, requiring incision and drainage to promote secondary healing[10].

Highly complex anal fistulas exhibit a complex anatomical structure owing to the fistula direction above the deep part of the sphincter as well as multiple internal openings and pipelines. Consequently, surgical treatments for these fistulas carry a high risk of damaging the anorectal ring, resulting in potential complications, such as anal incontinence or deformity, and even significantly affecting the patient’s quality of life[29]. The internal opening of high anal fistulas is usually situated at the posterior midline anal gland. Studies have shown that high posterior glandular anal fistulas mainly develop in the anal sphincter gap, which is a closed space bound by the internal and external sphincters. The small size of this space does not allow expansion, with even a small amount of pus accumulation potentially leading to high pressure[30]. The infection can also break through the posterior rectal space or ischiorectal fossa, forming an upper or transanal sphincter fistula[31]. Most sites of infection in highly complex anal fistulas are located in the DPIS. DPIS infections are closed infections that necessitate treatment with a complete opening of the closed lesion and maintenance of drainage. Anal MRI is more precise than fistulography in diagnosing anal fistulas and does not increase the risk of infection or induce other discomforts[32]. EAUS is extremely helpful in evaluating the fistula opening, fistula course, and sphincter defects[33]. Although EAUS is not as accurate as MRI, it offers advantages such as simplicity, cost-effectiveness, and reproducibility[34].

In recent years, numerous approaches have been employed to preserve the sphincter muscle in anal fistula surgery, including improvements in traditional surgical techniques and new innovative methods such as the more common clinical surgical methods of VAAFT, LIFT, and laser surgery. The VAAFT procedure, which was initially proposed and clinically applied in 2011 by Meinero *et al.*, was inspired by cystoscopy and focused on exploring the fistula internally via a fistuloscope introduced from the external fistula, enabling the identification of the correct internal fistula opening and performing electrocautery, scraping, and rinsing for further removal of necrotic tissue from the fistula[35]. However, VAAFT is not an effective intervention for intersphincteric abscesses and is associated with high costs and treatment difficulties for curved, stenotic, or horseshoe-shaped fistulas. Relevant studies have reported that the average recurrence rate after VAAFT is 18%[36]. Furthermore, VAAFT relies on specially designed equipment and the cost of this technique is currently unclear[37]. The LIFT method was first described in 2007 by Rojanasakul *et al*[38]. Compared with TROPIS, LIFT additionally involves the intersphincteric tract, wherein LIFT closes the intersphincteric tract endoprosthesis. Considerable skill is required for the accurate identification and complete debridement of fistulas during surgery for complex anal fistulas, especially recurrent and high-grade complex anal fistulas. Anal fistula surgery using the LIFT has been shown to cause less trauma, avoid sphincter damage, and lower the risk of fecal incontinence[39]. The failure rate of LIFT is about 28.6%[40]. Several refinements of the LIFT procedure have been explored, including bioprosthetic grafts to reinforce ligation of the intersphincteric fistula tract (BioLIFT)[41] and ligation of the intersphincteric fistula tract plus a bioprosthetic anal fistula plug (LIFT-Plug)[42], yielding cure rates as high as 94% and 95%, respectively. However, the high cost of these techniques prevents their widespread implementation in clinical settings. LIFT involves the ligation of the internal opening of the anal fistula. This approach effectively controls contamination by feces entering the fistula tract, promotes healing without requiring sphincter cutting, and increases the risk[9]. Similar to other biologics, BioLIFT incurs high costs, leading to an increased cost for operable procedures[37]. In addition to the aforementioned techniques, Bobkiewicz[43] proposed a new protocol for endoscopic vacuum therapy with instillation (iEVT) for the treatment of anal fistulas. iEVT combines the standard endoscopic vacuum therapy and negative-pressure wound therapy with instillation. This procedure is advantageous because it is minimally invasive and can be used as a transitional treatment before surgery. However, studies with larger sample sizes and long-term follow-up are needed to determine the efficacy of iEVT. Other surgical modalities for treating highly complex anal fistulas, such as expanded adipose-derived stem cells[44], dermal collagen injections[45], and protein binders[46], require further research to prove their effectiveness, with no clear literature currently available on their cure rates and surgical costs. In the 2022 edition of the American Society of Colon and Rectal Surgery Clinical Practice Guidelines, fistulotomies are recommended for the management of simple fistulas. Incision and hanging surgeries were also deemed safe and effective for fistulas; however, inevitable impairment of anal function was also noted. Moreover, fistula pessaries and fibrin glue filling are no longer considered first-line therapeutic options because of their low cure rates (50% or lower). Although VAAFT had reasonable short-term cure rates, no long-term follow-up data were available for these two procedures. Therefore, VAAFT is no longer recognized as a first-line therapeutic option[47]. Laser surgery is affected by the wavelength and energy of the laser, which makes it difficult to observe fistula contractions more intuitively and does not guarantee its effectiveness in the treatment of larger fistulas. It seems that it cannot meet the clinical treatment requirements[48].

Given that a lack of demonstrable heterogeneity may mask clinical heterogeneity, heterogeneity tests may reveal subgroup effects[49]. Hence, we tested for heterogeneity by examining the treatment of highly complex anal fistulas with surgery in terms of one-time cure rate, recurrence rate after the first surgery, and final cure rate. The results showed no heterogeneity among the included studies; consequently, a fixed-effects model was used for further analyses. The accuracy and stability of this study were ensured by conducting a sensitivity analysis. In this analysis, the stability of the results was assessed[50], revealing that none of the studies notably interfered with the findings of the current meta-analysis, highlighting data stability in our study. Publication bias was tested using funnel plots, Begg’s test, and Egger’s test, and the results established that the included studies were representative and the conclusions of the meta-analysis were stable[51]. All these results revealed that TROPIS had significant efficacy in treating highly complex anal fistulas.

## Conclusions

In this meta-analysis, we objectively investigated the effectiveness of TROPIS in treating high complex anal fistulas, focusing on the clinical situation of the patients who achieved surgical cure. Our results revealed that TROPIS treatment for these anal fistulas provides a high one-time cure rate, low recurrence rate, and elevated overall cure rate, with no observed adverse events. Therefore, TROPIS appears to be a safe and effective alternative surgical option for treating highly complex anal fistulas. However, this study has a few limitations that should be considered. Given that only six eligible studies were included, our study had a small sample size and limited number of patients. Therefore, future clinical studies with large samples and multicenter trials are warranted to validate the efficacy and safety of TROPIS in treating high complex anal fistulas. Additionally, the included literature was comprised of non-randomized controlled studies; thus, further randomized controlled trials are required to examine and verify the effectiveness of TROPIS in the treatment of these fistulas. The included studies did not conduct detailed quantitative evaluations such as the St. Mark’s incontinence score and quality of life questionnaires. These shortcomings should be addressed in future studies to demonstrate the effectiveness and safety of the TROPIS.

## Data Availability

The data that support the findings of this study are available from the corresponding author, Pengfei Zhou, upon reasonable request.
